# Gene expression profiling of upregulated mRNAs in granulosa cells of bovine ovulatory follicles following stimulation with hCG

**DOI:** 10.1186/s12958-017-0306-x

**Published:** 2017-11-03

**Authors:** Jacques G. Lussier, Mame N. Diouf, Valérie Lévesque, Jean Sirois, Kalidou Ndiaye

**Affiliations:** 1 Centre de recherche en reproduction et fertilité, Faculté de médecine vétérinaire, Université de Montréal, 3200 Sicotte, St-Hyacinthe, Québec, J2S 2M2 Canada; 2 Institut Sénégalais de Recherches Agricoles (ISRA) Laboratoire National de l′Elevage et de Recherches Vétérinaires (LNERV), BP 2057 Dakar-Hann, Sénégal

**Keywords:** Ovary, Follicle, Ovulation, Granulosa cells, Gene expression

## Abstract

**Background:**

Ovulation and luteinization of follicles are complex biological processes initiated by the preovulatory luteinizing hormone surge. The objective of this study was to identify genes that are differentially expressed in bovine granulosa cells (GC) of ovulatory follicles.

**Methods:**

Granulosa cells were collected during the first follicular wave of the bovine estrous cycle from dominant follicles (DF) and from ovulatory follicles (OF) obtained 24 h following injection of human chorionic gonadotropin (hCG). A granulosa cell subtracted cDNA library (OF-DF) was generated using suppression subtractive hybridization and screened.

**Results:**

Detection of genes known to be upregulated in bovine GC during ovulation, such as *ADAMTS1*, *CAV1*, *EGR1*, *MMP1*, *PLAT*, *PLA2G4A*, *PTGES*, *PTGS2*, *RGS2*, *TIMP1*, *TNFAIP6* and *VNN2* validated the physiological model and analytical techniques used. For a subset of genes that were identified for the first time, gene expression profiles were further compared by semiquantitative RT-PCR in follicles obtained at different developmental stages. Results confirmed an induction or upregulation of the respective mRNAs in GC of OF 24 h after hCG-injection compared with those of DF for the following genes: *ADAMTS9*, *ARAF*, *CAPN2*, *CRISPLD2*, *FKBP5*, *GFPT2*, *KIT*, *KITLG*, *L3MBLT3*, *MRO*, *NUDT10*, *NUDT11*, *P4HA3*, *POSTN*, *PSAP*, *RBP1*, *SAT1*, *SDC4*, *TIMP2*, *TNC* and *USP53*. In bovine GC, *CRISPLD2* and *POSTN* mRNA were found as full-length transcript whereas *L3MBLT3* mRNA was alternatively spliced resulting in a truncated protein missing the carboxy-terminal end amino acids, ^774^KNSHNEL^780^. Conversely, *L3MBLT3* is expressed as a full-length mRNA in a bovine endometrial cell line. The ^774^KNSHNEL^780^ sequence is well conserved in all mammalian species and follows a SAM domain known to confer protein/protein interactions, which suggest a key function for these amino acids in the epigenetic control of gene expression.

**Conclusions:**

We conclude that we have identified novel genes that are upregulated by hCG in bovine GC of OF, thereby providing novel insight into peri-ovulatory regulation of genes that contribute to ovulation and/or luteinization processes.

**Electronic supplementary material:**

The online version of this article (10.1186/s12958-017-0306-x) contains supplementary material, which is available to authorized users.

## Background

In cattle, ovarian follicular development is characterized by two or three consecutive follicular waves per estrous cycle. Each wave involves the recruitment of a cohort of follicles at the diameter of 3- to 4-mm. A selection phase follows, in which a single follicle continues its development and becomes the dominant follicle (DF), whereas subordinate follicles degenerate by atresia [1]. The DF of the final wave of the estrous cycle pursues its growth and matures into an ovulatory follicle (OF) following the release of the preovulatory LH surge. The ovulatory process begins at the time when the endogenous preovulatory LH surge stimulates G protein-coupled LH/human chorionic gonadotropin receptors (LHCGR) located on granulosa and theca cells. Activation of the LHCGR induces a developmental program in different compartments of the DF to ensure the release of a competent oocyte via changes in formation/organization of hyaluronan-rich cumulus extracellular matrix followed by rupture of follicular wall, and by formation of the corpus luteum through differentiation of granulosa and theca cells into luteal cells. These processes are controlled by the expression of many genes that are either up- or downregulated in a temporally and spatially distinct fashion. The mechanisms ensuring the transition from a DF into an OF are not fully understood [2]. This information is essential to advance our understanding of the cascade of events leading to ovulation and luteinization of the follicle, which may be further applied to enhance fertility.

Genes expressed in granulosa cells (GC) that control the growth of a bovine dominant or preovulatory follicle are rapidly downregulated as a consequence of post LH surge-mediated increases in intracellular signaling [3, 4]. In conjunction with the termination of specific gene expression in preovulatory follicles, LH/hCG induces expression of genes involved in ovulation and luteinization, as shown in rodents [5] or in the bovine species [4, 6, 7]. Most data on the temporal pattern of gene expression arising during ovulation have been obtained using RNA extracts from the immature eCG- and hCG-stimulated rodent ovaries. Moreover, the use of microarray in rodent or bovine studies are limited to genes present in the array, and results among bovine studies vary in relation to experimental design and treatment used [4, 6, 7]. The objective of the present study was to identify candidate genes that are upregulated in GC of bovine preovulatory follicle in response to an ovulatory stimulus. The working hypothesis was that the transition from a DF into an OF results from transcriptional induction or upregulation of a subset of genes in GC. Gene expression was studied in GC because they represent an important compartment of the ovarian follicle involved in hormone synthesis and maturation of the oocyte [2]. The GC were obtained from hCG-induced OF and growing DF collected during the first follicular wave of the bovine estrous cycle. Gene expression analysis was achieved by use of suppression subtractive hybridization (SSH) [3, 8] that results in enrichment of differentially expressed genes in OF, followed by the establishment of a GC subtracted cDNA library (OF-DF). The cDNA clones isolated from the subtracted library were validated for their differential expression pattern by cDNA macroarrays and characterized by sequencing. A subset of genes found to be differentially expressed by macroarrays were further validated by semiquantitative reverse transcriptase polymerase chain reaction (RT-PCR) performed on independent follicles and corpus luteum obtained at different developmental stage. Also, we undertook the characterization of the differentially expressed mRNA of cysteine-rich secretory protein LCCL domain-containing 2 *(CRISPLD2)*, periostin (*POSTN)* and lethal (3) malignant brain tumor-like protein (*L3MBTL3)* full-length cDNAs since they were not experimentally characterized in the bovine species.

## Methods

### Experimental animal model and sample preparations

Normal cycling crossbred heifers were synchronized with one injection of PGF_2α_ (25 mg, im; Lutalyse, Upjohn, Kalamazoo, MI) given in the presence of a corpus luteum (CL). Behavioral estrus was monitored at 12 h intervals, from 48 h to 96 h following the PGF_2α_ injection. From the time of PGF_2α_ injection until ovariectomy, ovarian follicular development was monitored by daily transrectal ultrasonography performed with a real-time linear scanning ultrasound diagnostic system (LS-300; Tokyo Keiki Co, Ltd., Tokyo, Japan) equipped with a 7.5-MHz transducer probe. At each examination, the diameter of the CL and individual follicles ≥ 4 mm were measured at their largest cross-sectional area using internal calipers. Following estrus synchronization by PGF_2α_, heifers were randomly assigned to the dominant follicle group (DF; *n* = 4) or the ovulatory hCG-induced follicle group (OF; n = 4). In the DF group, the ovary bearing the DF on the morning of day 5 of the estrous cycle (day 0 = day of estrus) was obtained by ovariectomy (via colpotomy). The DF was defined as >8 mm by ultrasonographic measurement and growing while subordinate follicles were either static or regressing [9]. The mean diameter of DF measured at the surface of the ovary was 10.4 ± 0.3 mm. The OF were obtained following an injection of 25 mg of PGF_2α_ (Lutalyse) on day 7 to induce luteolysis, thereby maintaining the development of the DF of the first follicular wave into a preovulatory follicle [10]. An ovulatory dose of hCG (3000 IU, iv; APL, Ayerst Lab, Montréal, QC) was injected 36 h after the induction of luteolysis, and the ovary bearing the hCG-induced OF was collected by ovariectomy at 24 h after hCG injection. The mean diameter of OF was 12.9 ± 0.3 mm. Follicular fluid and mural GC with whole cumulus-oocyte complex were collected separately from individual DF or OF as described previously [9]. Additionally, GC and follicular fluid were collected from 2 to 4 mm follicles that were obtained from slaughterhouse ovaries representing a total of three pools of 20 small follicles (SF). These experiments were approved by the Animal Ethics Committee of the Faculty of Veterinary Medicine of the Université de Montréal. Concentrations of progesterone (P_4_), estradiol-17β (E_2_) and their ratio (P_4_/E_2_) were analyzed by RIA of follicular fluid as previously described [9]. The E_2_/P_4_ ratios were calculated for each sample: 1) 0.008, 0.06 and 0.01 for the three SF pools; 2) 17.3, 19.7, 14.3 and 63.4 for individual DF at day 5 (*n* = 4); and 3) 0.44, 0.87, 0.64 and 0.27 (n = 4) for individual OF. CL at day 5 of the estrous cycle were obtained by ovariectomy from cows following ultrasound monitoring of follicular development and estrus synchronization as described above. The CL were dissected from the ovarian stroma, frozen in liquid nitrogen, and then stored at −80 °C until RNA extraction. Total RNA was isolated from GC or CL by homogenization in lysis buffer (4 M guanidium isothiocyanate, 0.5% Na-N-laurylsarcosine, 25 mM NaCitrate, pH 7), and total RNA sedimented on a cesium chloride cushion by ultracentrifugation as previously described [9]. The concentration of total RNA was quantified by measurement of optical density at 260 nm, and quality was evaluated by visualizing the 28S and 18S ribosomal bands following electrophoretic separation on a formaldehyde denaturing 1% agarose gel with ethidium bromide.

### Suppression subtractive hybridization and differential hybridization screening

To compare gene expression in GC collected from OF versus DF, the suppression subtractive hybridization (SSH) was performed as previously described [8]. Identical amounts of total RNA (2 μg) from four OF or four DF were pooled within treatment groups to decrease inter animal variation. To generate sufficient amounts of double-stranded cDNA for an SSH experiment, both OF and DF cDNAs were amplified separately using the SMART PCR cDNA synthesis kit. One microgram of total RNA from each pooled group was reverse transcribed with an oligo-dT30 primer [CDS: 5′-AAGCAGTGGTAACAACGCAGAGTACT(30)(A/C/G/T) (A/G/C)-3′] and PowerScript (BD Biosciences Clontech) to generate the first strand cDNA. Second cDNA strands were produced with the SMART II 5′-anchored oligo and PCR-amplified for 15 cycles using Advantage 2 DNA polymerase (BD Biosciences Clontech). PCR-generated OF and DF cDNAs were digested with *Rsa*I to generate blunt-ended cDNA fragments [from 0.2 to 2 kilobases (kb)]. The OF cDNAs were subtracted against DF cDNAs (forward reaction: OF-DF) using PCR-Select cDNA subtraction technology (User manual: PT1117-1; BD Biosciences Clontech) [8], and in a parallel experiment, the DF cDNAs were subtracted against the OF cDNAs (reverse reaction: DF-OF). The efficiency of subtraction was analyzed by comparing the abundance of cDNAs before and after subtraction by PCR using bovine gene-specific primers for a gene known to be induced by hCG, such as prostaglandin-endoperoxide synthase 2 (*PTGS2*), and a gene known to be downregulated by hCG, such as cytochrome P450, family 19, subfamily 1 (*CYP19A1*). PCR amplification was performed using Advantage 2 DNA polymerase (BD Biosciences Clontech), and 5-μl aliquots were removed following determined numbers of PCR cycles. The amplified products were resolved on a 2% agarose gel in TAE buffer (40 mM Tris-acetate, 1 mM EDTA, 0.5 μg/ml ethidium bromide). The difference in the number of cycles needed to generate an equal amount of the corresponding PCR product in subtracted and unsubtracted samples served to indicate the subtraction efficiency.

The subtracted cDNAs were cloned into the pT-Adv plasmid (BD Biosciences Clontech) to construct the OF-DF subtracted library and used to transform competent TOP10F’ *Escherichia coli* as previously described [11]. The subtracted OF-DF cDNA library (940 individual colonies) was used to establish macroarrays for differential screening following previously described methodologies [8]. The insert of each cDNA clone was amplified in 96-well plates by PCR (28 cycles) using the PCR-nested primers 1 and 2R and AmpliTaq DNA polymerase (Roche Molecular Systems Inc., Laval, QC). To establish the cDNA macroarrays, an aliquot of each amplification product was denatured in 0.3 M NaOH with 5% bromophenol blue, and 10 μl were vacuum transferred with a 96-well dot-blot apparatus onto nylon membranes (Hybond-N^+^, GE Healthcare Life Sciences), which were then exposed to 150 mJ ultraviolet light (UV) to perform DNA cross-linking (Gs Gene Linker; Bio-Rad, Mississauga, ON). Control cDNAs (*CYP19A1, PTGS2*) were transferred onto the macroarrays. For each 96-well plate, two identical cDNA macroarray replicate membranes were generated. The OF-DF and DF-OF cDNA pools were used to generate complex hybridization probes for differential screening of macroarrays of the OF-DF cDNA library. Probes were obtained by performing the secondary nested PCR and were then purified (QIAquick PCR Purification Kit; Qiagen Inc., Mississauga, ON). To prevent nonspecific interaction of the probes to cDNAs on macroarrays during hybridization, the adaptors were removed by three successive digestions with *Afa*I, *Sma*I, and *Eag*I; the cDNA pools were again purified (QIAquick; PCR Purification Kit, Qiagen Inc.) and 100 ng were labeled with [α^32^P]-dCTP by random priming (Megaprime DNA Labeling System; GE Healthcare Life Sciences). The radioactive probes were purified (QIAquick Nucleotide Removal kit; Qiagen Inc.) and quantified using a beta counter. The hybridization and washing conditions of macroarrays were performed as previously described [8]. Equal amounts of each heat-denatured cDNA probe (OF-DF, DF-OF) were used to hybridize each replicate of the OF-DF macroarray membrane. Following washing, membranes were exposed to a phosphor screen for 4 h and the images were digitized (Storm 840; GE Healthcare Life Sciences). The differentially hybridizing cDNA clones were characterized by DNA sequencing and their differential expression profiles were further validated by semiquantitative RT-PCR analysis from independent follicles obtained at different developmental stages.

Identification of differentially expressed cDNAs were obtained by sequencing. The cDNA clones identified as differentially expressed by the OF-DF subtracted probe were amplified by PCR for 15 cycles with the corresponding PCR-nested 1 and PCR-nested 2 oligos from the PCR product generated initially for the macroarrays. The PCR product was purified (Qiagen Inc.) and verified by agarose gel analysis for the presence of a single cDNA band before proceeding with sequencing. Sequencing reactions were performed on cDNA clones via the dideoxy sequencing method (Big Dye Terminator 3.0; ABI Prism, Applied BioSystem, PE, Branchburg, NJ) using the oligos PCR-Nested 1 or 2, and sequencing reactions were analyzed on an ABI Prism 310 sequencer (Applied Biosystems). Nucleic acid sequences were analyzed by BLAST (Basic Local Alignment Search Tool) against GenBank data banks.

### Gene expression analysis

The cDNA clones that were identified as differentially expressed in the SSH differential screening experiment were used to compare their differential expression pattern in GC collected from follicles at different developmental stages and CL, using semiquantitative RT-PCR/Southern blot analysis (also called virtual Northern blot analysis; User manual: PT1117-1; BD Biosciences Clontech). To perform semiquantitative RT-PCR/Southern blot analysis, total RNA (1 μg) from GC (SF, DF, OF) or CL (D5) were reverse transcribed using SMART PCR cDNA synthesis technology (BD Biosciences Clontech, Mississauga, ON) as previously described [8]. The cDNA products for each follicle or CL along with molecular weight standards (1 kb ladder, ϕX 174-RF/Hae III and l/Hind III; GE Healthcare Life Sciences) were separated on agarose gel, then transferred onto nylon membrane. Gene-specific probes derived from SSH cDNA fragments were generated by PCR (20 cycles) using the primers PCR Nested 1 and PCR-Nested 2R. Glyceraldehyde-3-phosphate dehydrogenase (*GAPDH*) probe was used as a constitutively expressed gene. Purified cDNA probes were labeled with [α^32^P]-dCTP as described above. Semiquantitative RT-PCR/Southern membranes were prehybridized, hybridized, and washed as described [8]. Membranes were exposed to phosphor screen and the images were digitized (Storm 840; GE Healthcare Life Sciences).

Semiquantitative RT-PCR analysis was performed for genes that showed a weak signal by RT-PCR/Southern blot or to analyze differentially spliced transcripts [8]. Gene-specific primers were designed into the open reading frame of the cDNA sequence and are described in Table 1. SMART cDNAs were generated as described above, and 1 μl was used in a 25-μl PCR reaction using the Advantage 2 DNA polymerase. The number of PCR cycles were limited and optimized to fall in the linear range of amplification reaction for each gene to be analyzed. The PCR reactions were separated on a 2% TAE-agarose gel with ethidium bromide, PCR products were visualized by UV, and the images digitized. The digitized signals for each gene obtained either by semiquantitative RT-PCR/Southern blot or semiquantitative RT-PCR were analyzed by densitometry using ImageQuant software (GE Healthcare Life Sciences).

**Table 1:** Gene-specific primers used in SSH and semiquantitative RT-PCR analysis

Gene		Primer Sequence (5′-3′)^a^	GenBank accession number
*ADAMTS1*	Fwd	CGATAAATGTGGCATCTGTGGAG	NM_001101080
	Rv	AGCCCACACGACTTGGAACACTC	
*ADAMTS9*	Fwd	GGAAATCCGTATTGGGAATGCTG	NM_001206573
	Rv	CATCTAGCCTGCTGTACTTGGC	
*ARAF*	Fwd	TCTAACAACATCTTCCTGCACGAG	NM_001014964
	Rv	GGCAACTCATCAGCCTGGGTAC	
*CAPN2*	Fwd	TGAGGGCTTCGAGGACTTCACTG	NM_001103086
	Rv	GCTGTCACTGGTGAGTGTGTCAG	
*CRISPLD2*	Fwd	CTATGGGATCCTGGACGACAGG	AY369781
	Rv	CCTGCAAGTTCACTGCCTGACG	
*CYP19A1*	Fwd	GTCCGAAGTTGTGCCTATTGCCAGC	NM_174305
	Rv	CCTCCAGCCTGTCCAGATGCTTGG	
*FKBP4*	Fwd	TCCATTCCTCGTGAATGAATGTCC	NM_001034322
	Rv	TGAACCTTCAGCAACGTGCAGAG	
*FKBP5*	Fwd	ACTGGAGCAGGCTGCCATTGTC	XM_615814
	Rv	CTTGAACATGTTGGCATAGATTCTG	
*GAPDH*	Fwd	TGTTCCAGTATGATTCCACCCACG	NM_001034034
	Rv	CTGTTGAAGTCGCAGGAGACAACC	
*KITLG1*	Fwd	AGTAACCGTGTGACTGATGATGTG	NM_174375
	Rv	AGAATGCTGGCAATGCTACGGCTG	
*KITLG2*	Fwd	AGTAACCGTGTGACTGATGATGTG	NM_174375
	Rv	CTAAGGGAGCTGGCTGCAACAG	
*L3MBTL3*	Fwd	GTTATTACAGATGAGAGTGAGATGG	AY437805
	Rv	ACTTAAAGGACAGAAAGACGGC	
*L3MBTL3*	Fwd	CTTACCTGGATGTGAAGAACATGG	AY437805
(3’end of ORF)	Rv	CTGCAAGGTCTAAGACAGAGCTC	
*MMP1*	Fwd	CTTGGACTTGCTCATTCTACTGAC	NM_174112
	Rv	GGCATCGATGCTCTTCACCGTTC	
*MRO*	Fwd	GGTGAGTTCTGAAGTCATCCATG	BC146146
	Rv	CCTGGTGTGCCGACCACCTTC	
*NUDT10*	Fwd	GACAGGTGAGCTCTTTCACACTC	NM_001035488
	Rv	GGAGTTATGTCTAGAGGCACAGTC	
*NUDT11*	Fwd	GAACAGCAAAGATGTCCAGGATTG	NM_001101296
	Rv	CACACACATGGTGCCTGGAGAG	
*P4HA3*	Fwd	TTGGCAAGGTGGCCTATGACATG	NM_001001598
	Rv	CTGGAGCAGCAGGTAAGGACTG	
*POSTN*	Fwd	CCAAAGCCCACTGCCAGTTCTC	AY445072
	Rv	GAAAGCCACTTTGATGTGAAGAATGAG	
*PSAP*	Fwd	CTTCCTCCTGTACCCTCAGGAC	NM_174161
	Rv	GGGATGACGTTCTCTGAGACCAC	
*PTGS2*	Fwd	GCATTCTTTGCCCAGCACTTCACCC	NM_174445
	Rv	CTATCAGGATTAGCCTGCTTGTCTGG	
*RBP1*	Fwd	CGGTCGACTTTACCGGGTACTG	NM_001025343
	Rv	GTCATGTCACTCATTCCTAGAGAC	
*SDC4*	Fwd	TCCGAGAAACTGAGGTCATCGAC	XM_584869
	Rv	GGTACACCAGCAGCAGCACGAG	
*TIMP1*	Fwd	CGTCATCAGGGCCAAGTTCGTG	NM_174471
	Rv	GCAAGGACTGCCAGGTGCACAG	
*TIMP2*	Fwd	TGCAATGCAGACATAGTGATCAGG	NM_174472
	Rv	CGCTTCTCTTGATGCAGGCGAAG	
*TNC*	Fwd	CTATGTGCCCATTGCAGGAGGTG	NM_001078026
	Rv	AACTTGGTGGTGATGGTTGAGCTC	
*USP53*	Fwd	GAGAGCATACCCACCAGTCAGATG	BC149007
	Rv	GTTACTTTGACATCAGGAGTAGAGTC	

*Fwd* foward primer, *Rv* Reverse primer

^a^All primers were designed and validated by the authors

### Characterization of bovine POSTN, CRISPLD2 and L3MBTL3 cDNAs

Isolation of full-length bovine periostin (*POSTN*), cysteine-rich secretory protein LCCL domain containing 2 (*CRISPLD2)* and lethal (3) malignant brain tumor-like protein (*L3MBTL3)* cDNAs was performed by screening a size-selected cDNA library for each of the cDNA as described [11], since their respective full-length cDNAs were not experimentally characterized in the bovine species. Initially, the respective sizes of the full-length cDNA were estimated by performing a semiquantitative RT-PCR/Southern blot analysis with cDNA pool generated from hCG-stimulated GC and hybridized with radioactive probes for *POSTN*, *CRISPLD2* or *L3MBTL3* derived from the SSH screening experiment (Table 2). Once the size of the full-length bovine *POSTN*, *CRISPLD2* and *L3MBTL3* cDNAs were determined, total SMART cDNAs from hCG-stimulated GC were size-fractionated by agarose gel electrophoresis, and cDNAs from 2.5 to 3.5 kb for *POSTN*, and from 4 to 6 kb for *CRISPLD2* and *L3MBTL3* were purified and used to construct a size-selected cDNA library based on the pDrive plasmid (Qiagen PCR cloning kit; Qiagen) that was then screened by radioactive hybridization as described [11]. Positive *POSTN, CRISPLD2* and *L3MBTL3* hybridizing bacterial colonies were grown, their plasmid contents were isolated (QIA-prep, Qiagen), and the size of the cloned cDNA was analyzed following an *EcoR1* digestion and gel electrophoresis analysis. The cDNAs were sequenced via the dideoxy sequencing method (Big Dye Terminator 3.0; ABI Prism, Applied BioSystems, PE) that were analyzed on an ABI Prism 310 sequencer (Applied Biosystems). Nucleic acid sequences were analyzed by BLAST against GenBank data banks.

**Table 2:** Genes found to be differentially expressed in bovine granulosa cells of OF compared to DF

Gene	Accession Number^a^	Freq^b^	Accession Number^c^	Identity (%)^d^	E value	Description^e^
*ACTG1*	EG565281	3	NM_001033618	100%	0	BT Actin gamma 1
*ADAMTS1*	GR508913	1	NM_001101080	100%	2e^−126^	BT ADAM metallopeptidase with thrombospondin type 1 motif 1
*APP*	EG565335	1	NM_001076796	100%	e^−107^	BT Amyloid beta precursor protein
*ARAF*	EG565327	4	NM_001014964	100%	0	BT A-Raf proto-oncogene. Serine/threonine kinase
*ASB9*	EG565323	2	AY438595	99%	4e^−179^	BT Ankyrin repeat and SOCS box-containing 9
*BIRC2*	EG565293	1	XM_015466434	100%	2e^−67^	BT Baculoviral inhibitor of apoptosis repeat containing 2
*CAPN2*	EG565332	1	NM_001103086	100%	8e^−57^	BT Calpain 2, (m/II) large subunit
*CAV1*	EG565338	20	AY823915	100%	0	BT Caveolin 1
*CD83*	EG565265	1	NM_001046590	99%	0	BT CD83 molecule
*CDC42SE2*	EG565271	1	NM_001102537	96%	2e^−180^	BT CDC42 small effector 2
*CRISPLD2*	EG565333	16	AY369781	100%	0	BT Cysteine-rich secretory protein LCCL domain containing 2
*CSRP3*	EG565284	1	NM_001024689	99%	3e^−165^	BT Cysteine and glycine-rich protein 3
*CTSK*	EG565324	1	NM_001034435	100%	5e^−168^	BT Cathepsin K
*DHTKD1*	EG565315	1	NM_001205838	100%	3e^−170^	BT Dehydrogenase E1 and transketolase domain containing 1
*EGR1*	EG565268	1	AY924307	98%	9e^−127^	BT Early growth response 1
*EIF4E3*	EG565311	1	NM_001102306	99%	e^−179^	BT Eukaryotic translation initiation factor 4E family member 3
*FKBP5*	EG565325	6	NM_001192862	100%	0	BT FK506-binding protein 5
*GEM*	EG565288	2	NM_001083732	100%	0	BT GTP binding protein overexpressed in skeletal muscle
*GFPT2*	EG565318	5	NM_001076883	99%	4e^−168^	BT Glutamine-fructose-6-phosphate transaminase 2
*GRIA3*	EG565320	3	NM_001206059	99%	2e^−166^	BT Glutamate ionotropic receptor AMPA type subunit 3
*KIT*	EG565345	1	XM_005207937	99%	7e^−110^	BT KIT proto-oncogene receptor tyrosine kinase
*KITLG*	EG565276	5	NC_007303	99%	0	BT KIT ligand
*L3MBTL3*	EG565310	2	AY437805	100%	7e^−73^	BT Histone methyl-lysine binding protein
*LRIG1*	EG565303	1	XM_002696962	100%	2e^−84^	BT Leucine-rich repeats and immunoglobulin-like domains 1
*MAOA*	EG565344	4	NM_181014	99%	2e^−109^	BT Monoamine oxidase A
*MMP1*	GR508914	3	NM_174112	100%	8e^−83^	BT Matrix metallopeptidase 1
*MORF4L1*	EG565343	1	NM_001035448	98%	4e^−38^	BT Mortality factor 4 like 1
*MRO*	EG565273	4	XM_005224181	100%	1e^−95^	BT Maestro
*NT5E*	EG565299	1	NM_174129	100%	0	BT 5′-Nucleotidase, ecto
*NUDT10*	GR508915	2	NM_001035488	100%	2e^−138^	BT Nudix hydrolase 10
*NUDT11*	EG565272	3	NM_001101296	100%	0	BT Nudix hydrolase 11
*P4HA3*	EG565269	2	NM_001001598	100%	1e^−96^	BT Propyl 4-hydroxylase subunit alpha 3
*PAPSS2*	EG565283	1	XM_583370	99%	0	BT 3′-Phosphoadenosine 5′-phosphosulfate synthase 2
*PIR*	EG565279	1	NM_001102358	100%	1e^−101^	BT Pirin (iron-binding nuclear protein)
*PLA2G4A*	EG565305	1	AY363688	100%	4e^−164^	BT Phospholipase A2 group IVA
*PLAT*	EG565294	8	NM_174146	99%	0	BT Plasminogen activator, tissue type
*POSTN*	EG565275	33	AY445072	100%	0	BT Periostin
*PSAP*	EG565278	2	NM_174161	99%	8e^−89^	BT Prosaposin
*PTGES*	EG565342	1	AY032727	100%	1e^−122^	BT Prostaglandin E synthase
*PTGS2*	EG565287	24	AF031698	100%	0	BT Prostaglandin-endoperoxide synthase 2
*PTX3*	EG565317	2	NM_001076259	98%	3e^−61^	BT Pentraxin
*RBP1*	EG565267	1	NM_001025343	99%	5e^−101^	BT Retinol binding protein 1
*RGS2*	EG565346	4	NM_001075596	100%	7e^−130^	BT Regulator of G-protein signaling 2
*RND3*	EG565300	1	NM_001099104	100%	1e^−127^	BT Rho family GTPase 3
*SAT1*	EG565349	26	NM_001034333	99%	0	BT Spermidine/spermine N1-acetyltransferase 1
*SDC4*	EG565289	3	XM_584869	99%	1e^−136^	BT Syndecan 4
*SEH1L*	EG565340	1	NM_001103089	99%	1e^−158^	BT SEH1-like nucleoporin
*SLC25A17*	EG565266	1	NM_001045948	99%	0	BT Solute carrier family 25 member 17
*SOX4*	EG565285	1	NC_007324	99%	8e^−173^	BT SRY-box 4
*SULT1A1*	EG565308	1	NM_177521	99%	2e^−99^	BT Sulfotransferase family 1A member 1
*SULT1E1*	GR508916	2	NM_177488	99%	4e^−60^	BT Sulfotransferase family 1E member 1
*TIMP1*	GR508917	3	NM_174471	100%	2e^−90^	BT TIMP metallopeptidase inhibitor 1
*TIMP2*	EG565302	1	NM_174472	99%	5e^−163^	BT TIMP metallopeptidase inhibitor 2
*TMEM176B*	EG565301	1	NM_001099145	99%	3e^−165^	BT Transmembrane protein 176B
*TMSB4X*	EG565291	3	NM_001113231	99%	5e^−173^	BT Thymosin beta 4, X-linked
TMSB10	EG565264	1	NM_174623	100%	2e^−131^	BT Thymosin beta 10
*TNC*	EG565326	2	NM_001078026	99%	0	BT Tenascin C
*TNFAIP6*	EG565319	5	NM_001007813	99%	0	BT Tumor necrosis factor alpha-induced protein 6
*USP53*	EG565304	1	XM_015463685	100%	8e^−78^	BT Ubiquitin specific peptidase 53
*VNN2*	EG565337	2	NM_001163920	100%	0	BT Vanin 2
EST	EG565334	1	AC_000158	100%	3e^−74^	BT transcribed locus, chromosome 1
EST	EG565313	8	AC_000159	99%	3e^−143^	BT transcribed locus, chromosome 2 [3’UTR of TNFAIP6]
EST	EG565321	4	AC_000159	100%	0	BT transcribed locus, chromosome 2 [intron 1 of TNFAIP6]
EST	EG565339	2	AC_000159	99%	0	BT transcribed locus, chromosome 2 [intron 1 of TNFAIP6]
EST	EG565316	1	AC_000159	99%	0	BT transcribed locus, chromosome 2 [intron 1 of TNFAIP6]
EST	EG565341	2	AC_000159	99%	4e^−70^	BT transcribed locus, chromosome 2 [3’UTR of TNFAIP6]
EST	EG565336	1	AC_000161	99%	0	BT transcribed locus, chromosome 4 [3’UTR of Ubiquitin-conjugated enzyme E2H; UBE2H]
EST	EG565296	1	AC_000163	100%	0	BT transcribed locus, chromosome 6
EST	EG565307	1	AC_000167	98%	4e^−106^	BT transcribed locus, chromosome 10 [intron 2 of Nidogen 2; NID2]
EST	EG565328	1	AC_000169	99%	0	BT transcribed locus, chromosome 12 [intron 1 of Glypican-6; GPC6]
EST	EG565274	1	AC_000169	99%	3e^−133^	BT transcribed locus, chromosome 12
EST	EG565270	1	AC_000172	99%	8e^−175^	BT transcribed locus, chromosome 15 [3’UTR of Rho GTPase activating protein 20; ARHGAP20]
EST	EG565314	1	AC_000179	100%	6e^−156^	BT transcribed locus, chromosome 22 [intron 28 of ADAM metallopeptidase with thrombospondin type 1 motif 9; ADAMTS9]
EST	EG565295	1	AC_000180	100%	0	BT transcribed locus, chromosome 23 [intron of runt related transcription factor 2; RUNX2]
EST	EG565309	1	AC_000185	99%	0	BT transcribed locus, chromosome 28 [3’UTR of Sterile alpha motif domain containing 8; SAMD8]

^a^GenBank accession numbers of differentially expressed bovine SSH cDNA clones

^b^ *Freq* Frequency of cDNA clone identified from macroarray analyses of OF-DF subtracted library

^c^Accession number of the best match found following nucleotide sequence comparison via BLAST search in GenBank

^d^Identity (%) represents homology estimates of bovine SSH cDNA fragments with nucleotide sequences in GenBank via BLAST search

^e^ *BT Bos taurus*, *EST* expressed sequence tag, *UTR* untranslated region

### Statistical analysis

Gene-specific signals generated for semiquantitative RT-PCR/Southern blot and semiquantitative RT-PCR analyses were normalized with corresponding *GAPDH* signals for each sample. Homogeneity of variance between follicular groups and CL was verified by O’Brien and Brown-Forsythe tests. Corrected values of gene-specific mRNA levels were compared between follicular and CL groups by one-way ANOVA. When ANOVA indicated significant differences (*P* < 0.05), multiple comparisons of individual means for SF, DF, OF and CL groups were compared by the Tukey-Kramer test (*P* < 0.05). Pearson’s correlation (*P* < 0.05) analysis was used to compare amplicon levels for KITLG1 and KITLG2. Data were presented as least-square means ± SEM. Statistical analyses were performed using JMP software (SAS Institute, Inc.).

## Results

### Identification of differentially expressed genes by SSH

A cDNA library containing transcripts that are upregulated by hCG in GC was constructed by subtracting DF cDNAs from OF cDNAs (OF-DF). Reverse subtraction was also performed as a control and consisted of OF cDNAs subtracted from DF cDNAs (DF-OF). PCR amplification analysis was used to verify the efficiency of the subtraction procedure by comparing the expression of reference genes such as *PTGS2* in OF and *CYP19A1* in DF, before and after subtraction. In the OF sample, the *PTGS2* PCR product was observed after 18 cycles but was undetectable in the DF sample. In the OF-DF subtracted sample, the PCR-amplified *PTGS2* fragment was detected after 13 cycles, revealing that *PTGS2* cDNA had been efficiently enriched in the subtracted OF-DF sample when compared with unsubtracted OF sample, confirming the effectiveness of the subtraction (validation of subtraction is presented in a Additional file 1: Figure S1). Subtracted cDNAs were then cloned into plasmid vector to generate the OF-DF cDNA library. Differential hybridization screening was performed on the randomly selected bacterial colonies to eliminate false-positive clones. Colonies were spotted onto two identical sets of macroarrays, and the OF-DF or DF-OF subtracted cDNA preparations were used as probes to hybridize macroarrays. On each membrane, *PTGS2* and *CYP19A1* cDNAs were spotted as controls. Selection of differentially expressed cDNA clones was achieved by comparing signal intensities between the two macroarrays as defined by the following criteria. Positive clones hybridized with the OF-DF subtracted probes but not with the reverse subtracted probes, DF-OF. Representative differential screening results are illustrated in Additional file 1: Figure S2). Of the initial 940 clones, differential screening identified 279 positives as defined by the selection criteria based on comparing differential intensity of the signal. After visualization on agarose gels of PCR products derived from these clones followed by their sequencing, 261 clones generated sequencing results of adequate quality to be analyzed by BLAST against GenBank databases. Table 2 lists all the compared sequences with an example for each entry that was deposited in GenBank, as well as their frequency of identification by differential screening of the OF-DF library. This comparison revealed that 89.7% (234/261) corresponded to 60 non-redundant known genes, and 10.3% (27/261) corresponded to express sequence tags (EST). The BLAST analysis of these ESTs against the bovine genome revealed that 24 ESTs were associated with transcribed loci, either introns or untranslated regions (UTR) of mRNA, of defined genes as indicated in brackets for each EST (Table 2) whereas three ESTs were considered novel transcribed loci.

### Analysis of mRNA expression

**Fig. 1:**
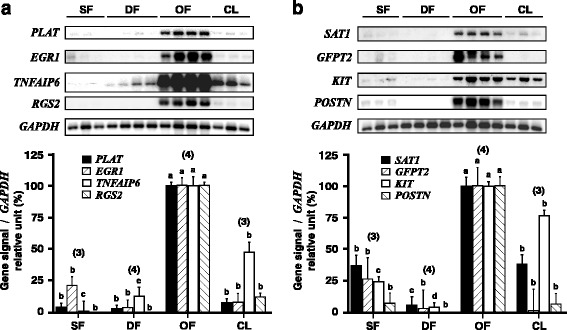
Analysis of mRNA expression by semiquantitative RT-PCR/Southern blot. Total RNA was extracted from bovine GC collected from 2 to 4 mm small follicles (SF), dominant follicles (DF) at Day 5 of the estrous cycle, ovulatory follicles (OF) collected 24 h after injection of hCG, and corpora lutea (CL) from Day 5 of the estrous cycle, then used in mRNA expression analyses using semiquantitative RT-PCR/Southern blot analysis. *GAPDH* was used as a control gene and showed no significant difference in expression between groups. Gene-specific signals were normalized with corresponding *GAPDH* signals (1.8 kb) for each sample, and relative values are reported as percent of expression detected in OF. **a**) Expression of *PLAT* (2.5 kb) mRNAs was upregulated by 32.8-fold in OF compared with DF (*P* < 0.0001); expression of *EGR1* mRNA (2.4 kb) was 24.7-fold higher in OF than in DF (*P* < 0.0001); expression of *TNFAIP6* mRNA (1.6 kb) was upregulated by 7.9-fold in OF compared with DF (*P* < 0.0001); and expression of *RGS2* mRNA (1.8 kb) was induced in OF compared with DF (*P* < 0.0001); **b)** expression of *SAT1* (2.5 kb) mRNAs was upregulated by 18-fold in OF compared with DF (*P* < 0.0001); expression of *GFPT2* mRNA (3 kb) was 26.8-fold higher in OF than in DF (*P* < 0.0029); expression of *KIT* mRNA (5 kb) was upregulated by 23.6-fold in OF compared with DF (*P* < 0.0001); and expression of *POSTN* mRNA (3.7 kb) was induced in OF compared with DF (P < 0.0001). Probability values for each one-way ANOVA analysis are specified above in parentheses. Different letters denote samples that differed significantly (*P* < 0.05) between group means for a specific gene. Data are presented as least-square means ± SEM, and the number of independent samples per group is indicated in parentheses

Genes identified by macroarray analysis as upregulated in GC following stimulation by hCG were further validated on the basis of their frequency of identification in the OF-DF subtracted library (Table 2). Validation was performed initially by semiquantitative RT-PCR/Southern blot analysis using mRNA samples derived from GC collected from independent follicles at different developmental stages (SF, DF, OF) and CL obtained at Day 5 of the estrous cycle. For these analyses, mRNAs were reverse transcribed into cDNAs, separated on agarose gel, and transferred onto membranes that were then hybridized to cloned SSH cDNA fragments as described in Table 2. Messenger RNA expression for tissue plasminogen activator (*PLAT*), early growth response 1 (*EGR1*), tumor necrosis factor alpha-induced protein 6 (*TNFAIP6*), regulator of G-protein signaling 2 (*RGS2*), spermidine/spermine N1-acetyltransferase 1 (*SAT1*), glutamine-fructose-6-phosphate transaminase 2 (*GFPT2*), KIT proto-oncogen receptor tyrosine kinase (*KIT*) and periostin (*POSTN*), showed a statistically significant increase or induction by hCG in GC of OF (Fig. 1). When mRNA signals observed for GC of OF were compared with that of DF, expression of *RGS2* and *POSTN* were induced, whereas for other genes, the increase was 32.8-fold for *PLAT*, 24.7-fold for *EGR1,* 7.9-fold for *TNFAIP6*, 18-fold for *SAT1*, 26.8-fold for *GFPT2* and 23.6-fold for *KIT*.

**Fig. 2:**
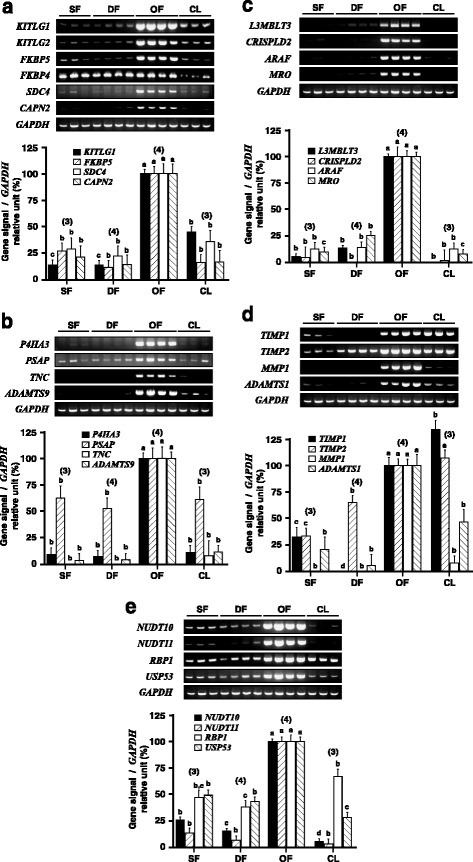
Analysis of mRNA expression by semiquantitative RT-PCR. Total RNA was extracted from bovine GC collected from 2 to 4 mm small follicles (SF), dominant follicles (DF) at Day 5 of the estrous cycle, ovulatory follicles (OF) 24 h after injection of hCG (OF), and corpora lutea (CL) from Day 5 of the estrous cycle, then used in mRNA expression analyses using RT-PCR. *GAPDH* was used as a control gene and showed no significant differences in expression between groups. Gene-specific RT-PCR amplicons were normalized with corresponding *GAPDH* (727 bp) amplicons for each sample. **a)** *KITLG1* (587 bp) and *KITLG2* (496 bp) mRNA were shown to be upregulated each by 6.7-fold in OF compared with DF (P < 0.0001); expression of *FKBP5* (450 bp) mRNA was increased by 8.6-fold in OF compared with DF (P < 0.0001); *FKBP4* (416 bp) mRNA did not differ between follicular stages but was reduced by 2.8-fold in CL when compared to OF (P < 0.0001); expression of *SDC4* (443 bp) mRNA was increased by 4.5-fold in OF compared to DF (*P* < 0.0005); and expression of *CAPN2* (453 bp) mRNA was increased by 7.1-fold in OF compared to DF (*P* < 0.0002); **b)** *P4HA3* (446 bp) mRNA was upregulated by 13.6-fold in OF compared to DF (P < 0.0001); *PSAP* (356 bp) mRNA was increased by 1.9-fold (*P* < 0.033); *TNC* (419 bp) mRNA was induced in OF compared to DF (P < 0.0002); and expression *ADAMTS9* (454 bp) mRNA was increased by 24.1-fold in OF compared to DF (P < 0.0001); **c)** *L3MBLT3* (1825 bp) mRNA was upregulated by 7.5-fold in OF compared to DF (P < 0.0001); *CRISPLD2* (493 bp) mRNA was induced in OF compared to SF and DF (P < 0.0001); *ARAF* (489 bp) mRNA was upregulated by 7.2-fold in OF compared to DF (P < 0.0001); and *MRO* (474 bp) mRNA was increased by 4-fold in OF compared to DF (P < 0.0001); **d)** *TIMP1* (487 bp) was induced in OF compared to DF (P < 0.0001); *TIMP2* (477 bp) was increased by 1.5-fold in OF compared to DF (P < 0.0001); *MMP1* (467 bp) was induced in OF compared to DF and SF (P < 0.0001); and *ADAMTS1* (482 bp) was upregulated by 18-fold in OF compared to DF (P < 0.0001); **e)** *NUDT10* (569 bp) was increased by 6.5-fold in OF compared to DF (P < 0.0001); *NUDT11* (466 bp) was upregulated by 15.2 fold in OF compared to DF (P < 0.0001); *RBP1* (444 bp) was increased by 2.6-fold in OF compared to DF (P < 0.0001); *USP53* (435 bp) was upregulated by 2.3-fold in OF compared to DF (P < 0.0001). Probability values for each oneway ANOVA analysis are specified above in parentheses. Different letters denote samples that differed significantly (P < 0.05) between group means for a specific gene. Data are presented as least-square means ± SEM, and the number of independent samples per group is indicated in parentheses

A semiquantitative RT-PCR assay was performed on selected genes described in Table 2 to confirm and compare their mRNA differential expression pattern in GC of hCG-induced OF. For this procedure, cDNAs were generated from total RNA, and the number of PCR cycles was optimized for each gene. All of the genes analyzed in this manner showed a statistically significant increase in hCG-treated GC of OF compared to that of DF (Fig. 2). Messenger RNA expression for KIT ligand (*KITLG1)* and isoform 2, *KITLG2*, each showed a 6.7-fold increase in GC of OF compared to that of DF (Fig. 2a). Intensity of amplicon signals obtained for *KITLG1* and *KITLG2* were highly correlated (*r* = 0.956). The FK506 binding protein 5 (*FKBP5*) mRNA was mainly observed in OF with a 8.6-fold increase in expression when compared to DF. Conversely, FK506 binding protein 4 (*FKBP4*) used as control, showed no difference in expression between follicular stages but was reduced by 2.8-fold in CL when compared to OF. Syndecan 4 (*SDC4*) transcript was mainly expressed in GC of OF wherein a 4.5-fold higher expression level was observed compared to DF. Calpain 2 (m/II) large subunit (*CAPN2*) transcript was detected in all groups and showed a 7.1-fold higher expression level in GC of OF compared to DF. The prolyl 4-hydroxylase subunit alpha 3 (*P4HA3*) mRNA was mainly observed in OF compared to other groups, and showed a 13.6-fold increase in OF compared to DF (Fig. 2b). Prosaposin (*PSAP*) mRNA was slightly increased by 1.9-fold in OF when compared to DF. Tenascin C (*TNC*) was induced in GC of OF when compared to DF and SF. ADAM metallopeptidase with thrombospondin type 1 motif 9 (*ADAMTS9*) found as an EST corresponding to intron 28 (Table 2) was upregulated by 24.1-fold in OF compared to DF. Expression of the histone methyl-lysine binding protein (*L3MBLT3*) mRNA showed an increase of 7.5-fold in OF compared to DF whereas no expression was detected in CL (Fig. 2c). Cystein-rich secretory protein LCCL domain containing 2 (*CRISPLD2*) mRNA was induced in OF when compared to DF. A-Raf proto-oncogene serine/threonine kinase (*ARAF*) mRNA was upregulated by 7.2-fold in OF compared to DF, and maestro (*MRO*) mRNA was increased by 4-fold in OF compared to DF. TIMP metallopeptidase inhibitor 1 (*TIMP1)* was induced in GC of OF when compared to DF whereas TIMP metallopeptidase inhibitor 2 (*TIMP2*) was upregulated by only 1.5-fold (Fig. 2d). Matrix metallopeptidase 1 (*MMP1*) was induced in GC of OF when compared to SF and DF, and was reduced in CL. ADAM metallopeptidase with thrombospondin type 1 motif 1 (*ADAMTS1*) was upregulated by 18-fold in OF compared to DF. Expression of nudix hydroylase 10 (*NUDT10*) and nudix hydroxylase 11 (*NUDT11)* mRNAs where upregulated by 6.5-fold and 15.2-fold, respectively, reaching the lowest level in CL (Fig. 2e). Retinol-binding protein 1 (*RBP1*) mRNA was upregulated by 2.6-fold in OF, and ubiquitin specific peptidase 53 (*USP53*) was upregulated by 2.3-fold.

### Characterization of bovine POSTN, CRISPLD2 and L3MBTL3 cDNAs

Bovine full-length cDNAs for *POSTN, CRISPLD2* and *L3MBTL3* cDNAs were not previously experimentally characterized in the bovine species. Since only cDNA fragments were obtained from the screening of the OF-DF subtracted library and that potential alternatively spliced isoforms may be expressed, we undertook their characterization in GC to ascertain the identity of the OF-DF cDNA fragments. Bovine cDNA fragments for *POSTN*, *CRISPLD2* and *L3MBTL3* were cloned from the SSH screening of the OF-DF subtracted library as presented in Table 1, and were used as probes to screen by hybridization size-selected cDNA generated from bovine GC that were collected 24 h following injection of hCG. The bovine *POSTN* cDNA consists of 3072 bp (Genbank: AY445072), and is composed of a 5′-UTR of 42 bp, an ORF of 2511 bp and a 3′-UTR of 519 bp containing two polyadenylation signals followed by a poly(A)^+^ tail. The coding region of bovine *POSTN* cDNA encodes a protein of 836 amino acids, with a theoretical molecular mass (Mr) of 93.1 kDa and an isoelectric point (pI) of 7. Amino acid homology analysis by PsiBlast revealed an overall identity level of 95% to human (NP_001129406). Bovine POSTN possesses a peptide signal (M^1^-A^21^) that may be cleaved between A^21^-N^22^, a cystein-rich domain called EMI domain (G^40^-A^94^), and four fasciclin domains (T^111^-T^231^, E^246^-P^367^, Q^381^-P^494^, R^508^-P^630^). Since five *POSTN* isoforms were reported in human, we verified by RT-PCR and sequencing if other *POSTN* isoforms were expressed in GC using primers targeting the entire ORF (Table 1). A single full-length cDNA of 2511 pb was characterized.

The bovine *CRISPLD2* cDNA consists of 4020 bp (Genbank: AY369781), and is composed of a 5′-UTR of 210 bp, an ORF of 1488 bp, and a 3′-UTR of 2322 bp containing six AU-rich elements (ATTTA) as well as two polyadenylation signals followed by a poly(A)^+^ tail. The coding region of *CRISPLD2* cDNA encodes a protein of 496 amino acids, with a theoretical Mr. of 55.6 kDa, pI of 8.2, and an overall identity level of 83% to human (NP_113664) and 76% to mouse (NP_084485) proteins. Bovine CRISPLD2 protein possesses a peptide signal (M^1^-G^22^) that may be cleaved between G^22^-F^23^, a cystein-rich secretory protein, antigen 5, and pathogenesis-related protein (known as CAP domain; L^60^-Y^200^) and two Limulus factor C, Coch-5b2 and Lgl1 domains (LCCL-1: V^287^-F^378^; LCCL-2: L^388^-T^482^).

The bovine *L3MBTL3* cDNA characterized from GC consists of 3892 bp (Genbank: AY437805), and is composed of a 5′-UTR of 205 bp, an open reading frame (ORF) of 2322 bp and a 3′-UTR of 1570 bp containing three AU-rich elements (ATTTA) as well as two polyadenylation signals followed by a poly(A)^+^ tail. The coding region of bovine *L3MBTL3* cDNA encodes a protein of 773 amino acids, with a theoretical Mr. of 87.5 kDa, pI of 6, and an overall identity level of 96% to human (GenBank: NP_115814) and 87% to mouse (NP_766375) proteins. Since the bovine protein expressed in GC was missing the last seven amino acids at its carboxy-terminal end, corresponding to ^774^KNSHNEL^780^ when compared to human protein, we verified by RT-PCR and sequencing if other *L3MBTL3* isoforms would be expressed in GC by using primers targeting the 3′-end of the open reading frame (Table 1). In parallel, RT-PCR analysis was performed using RNA extracted from a bovine endometrial epithelial and glandular cell line (Endo 8.3; [12]. In GC, a single alternatively spliced transcript was observed coding for the 773 amino acids protein that we previously characterized using the size selected cDNA library screening technique. This truncated *L3MBTL3* isoform is referred as transcript 2 (GenBank: AY437805). Conversely, a single transcript was also observed in the endometrial cell line that corresponded to the complete protein of 780 amino acids with a theoretical Mr. of 88.3 kDa and pI of 6.1, referred as *L3MBTL3* transcript 1 (GenBank: KT881243). Bovine L3MBTL3 protein isoforms expressed in GC and the endometrial cell line possesses three malignant brain tumor (MBT) repeat domains (MBT-1: M^268^-K^336^; MBT-2: M^375^-Y^442^; MBT-3: M^479^-P^543^), C2HC zinc finger domain (G^557^-S^586^) and a sterile alpha motif domain (SAM; S^706^-K^770^).

## Discussion

The preovulatory LH surge from the pituitary gland activates LH receptors on GC and theca cells, and induces a cascade of events in the dominant preovulatory follicle. They include the formation of the expanded hyaluronan-rich cumulus oophorus extracellular matrix (ECM), the release of a competent oocyte following the degradation/rupture of the follicular wall’s ECM, and the differentiation of GC and theca cells into luteal cells that contribute to corpus luteum formation. These processes are controlled by the expression of several genes that are either up- or downregulated in a temporally and spatially distinct fashion. However, the molecular mechanisms regulating the developmental transition of a dominant preovulatory follicle into an ovulatory follicle are not fully understood [2]. Identifying the precise subset of genes involved in these processes is essential to fully comprehend the cascade of events leading to ovulation and luteinization of the follicle, and will likely contribute to a better control of fertility.

We elected to use the SSH method for the identification of differentially expressed genes. This approach involved the screening by hybridization of the OF-DF subtracted GC cDNA library, which first allowed the selection of cDNAs based on differences in their signal intensities, and then their identification by DNA sequencing and further validation by semiquantitative RT-PCR. The genes upregulated in GC by hCG and identified by SSH in this study corroborate well those identified by microarray using a physiological model of endogenous LH release [4]. Screening the OF-DF subtracted cDNA library allowed identification of genes such as ADAMTS1 [13, 14], EGR1 [15], MMP1 [16], PLAT [16–18], PTGES [19], PTGS2 [10], PTX3 [4], TIMP1 [16], TIMP2 [20] and TNFAIP6 [21] that had previously been shown to be differentially expressed in GC of bovine ovulatory follicles compared to that of dominant preovulatory follicles. The identification of these genes validates the physiological model and the analytical approach used herein. Moreover, from the list of genes identified in Table 2, we also have previously investigated and confirmed the spatiotemporal induction of PLA2G4A [22] and CAV1 [23] at the mRNA and protein levels, and of VNN2 [24] and RGS2 [25] at the mRNA level, in GC collected at 0, 6, 12, 18, and 24 h post-hCG injection.

Of particular interest, FKBP5 and FKBP4 proteins, also known as FKBP51 and FKBP52, are nuclear receptor co-chaperones regulating cellular trafficking and activity of steroid hormone receptors such as the progesterone and glucocorticoid receptors. These co-chaperones compete for a common binding site on the heat shock protein 90 (Hsp90) that is complexed to steroid receptors. The level of FKBP4 and FKBP5 complexed to Hsp90 is determined by the abundance and affinity of each co-chaperone. FKBP5 is found in greater amount complexed to the progesterone receptor, as compared to FKBP4, and FKBP5 reduces, whereas FKBP4 increases, the binding affinity of the progesterone receptor toward its natural ligand [26, 27]. Interestingly, the presence of high levels of FKBP5 protein in various tissues of squirrel monkey primates has been identified as a potential cause of progesterone resistance in this species [28]. In the present study, we show that the expression of FKBP4 mRNA in GC does not differ in follicles larger than 2 mm up to ovulation. FKBP4 may potentiate the action of the progesterone receptor in GC since knockout mice for FKBP4 have a reduced number of ovulation [29]. Conversely, expression of FKBP5 mRNA increased in GC of hCG-induced ovulatory follicles. Since progesterone concentration increases in follicle following the LH/hCG preovulatory surge, and progesterone stimulates the transcription of FKBP5 [30], the increase of FKBP5 expression in bovine GC following the LH/hCG surge may represent a short negative feedback loop involved in attenuating GC progesterone responsiveness. Likewise, FKBP5 as a scaffolding protein was recently shown to recruit the phosphatase PHLPP to facilitate dephosphorylation of AKT and its downstream target p38MAPKα (also known as MAPK14), which in turn decreases activation of the glucocorticoid receptor [31–33]. A similar mechanism may be associated with the progesterone receptor in GC after the LH/hCG surge. Indeed, it is known that phosphorylation of AKT/p38MAPKα is increased in GC by the activation of the EGFR following binding of the EGF-like proteins, epiregulin (EREG) and amphiregulin (AREG) [34], and that EREG and AREG are induced in bovine GC after the LH/hCG surge [35, 36]. Such EGFR-dependent activation of the AKT/p38MAPKα pathway [34, 36] likely contributes to the phosphorylation and activation of the progesterone receptor [37]. Based on these observations and our findings, we hypothesize that the FKBP5 increase in bovine GC following the preovulatory LH surge may limit the EGFR-dependent AKT/p38MAPKα phosphorylation, and thus reduce progesterone receptor phosphorylation and activation, thereby attenuating GC progesterone responsiveness during ovulation and initial luteinization. The demonstration of such regulatory mechanism will require further investigations.

In addition to the list genes whose expression and/or role in ovulation has been in part or clearly determined, we have reported the cDNA characterization and the increased expression of POSTN, CRISPLD2 and L3MBTL3 mRNA in hCG-induced OF, and have conceived their possible function in the ovulatory follicle from what is known in the literature. We found that *POSTN* mRNA was induced by hCG in GC of OF but was absent in DF, in keeping with findings by Christenson et al. [4]. Only a single full-length *POSTN* mRNA was observed in hCG-stimulated bovine GC in the present study, as compared to the five *POSTN* isoforms identified in human (review: [38]). The possibility that POSTN could undergo post-translational modifications in GC will require further investigation [39]. POSTN is a secreted modular glycoprotein that interacts with various proteins of the ECM, growth factors and integrins. As an ECM protein, POSTN acts as an adhesive protein, binding to collagen I-V and fibronectin through its EMI domain, and to tenascin-C (TNC) through its FAS1 domains, to establish an interlaced ECM architecture. Thus, POSTN contributes to fibrillogenesis during normal tissue development, and tissue remodeling associated with various diseases, wound repair, inflammation and cancer [38]. Interestingly, we observed a concomitant increase in *TNC* and *POSTN* mRNAs in GC at ovulation, a coordinated expression also shown to be induced by mechanical stress in fibroblasts [40]. POSTN is known to interact with TNC, a complex disulfide-linked hexameric glycoprotein, to promote its incorporation to other ECM proteins such as collagen, fibronectin and heparan sulfate glycosaminoglycans [41]. POSTN is also recognized as a matricellular protein, a class of ECM proteins that promote non-structural roles such as cell adhesion, migration, proliferation, differentiation and survival [38, 39]. For these functions, POSTN interacts with integrins, that are the major membrane receptors mediating adhesion to the ECM, which lead to intracellular activation of the PI3K/AKT and focal adhesion kinase signaling pathways [38, 42, 43]. Such functions and activation could be present in GC since GC do express integrins [44]. Lastly, the ability of POSTN to induce angiogenesis by increasing VEGF receptor (KDR) expression in endothelial cells [43] may also be relevant to the marked neovascularization observed during the early stage of corpus luteum formation. Thus, the LH/hCG-dependent induction of *POSTN* mRNA in GC suggests its potential role as a key factor involved in various functions associated with ovulation, including GC survival, migration and luteinization, as well as angiogenesis and tissue remodeling required in the formation of the corpus luteum.

The role of CRISPLD2 in the female reproductive tract remains largely unexplored and, to our knowledge, this study is the first to document the hCG-dependent induction of *CRISPLD2* mRNA in GC of OF. CRISPLD2 is a member of the CAP (Cysteine-rich secretory proteins, Antigen 5, and Pathogenesis-related 1 proteins) superfamily of proteins, and is also known as CAPLD2. It is a secreted protein containing LCCL tandem domains that have been associated with multiple functions such as cell differentiation, migration, inflammation and immunity (review:[45]). CRISPLD2 expression has been involved in lung morphogenesis [46], kidney development [47], and in craniofacial morphogenesis [48]. Recently, it was shown to regulate proliferation, apoptosis, and migration of fetal lung fibroblasts, to contribute to mesenchymal-epithelial signaling, and to enhance wound repair [46]. In the latter study, CRISPLD2 was associated with the abnormal expression of multiple extracellular matrix (ECM) genes that modulate lung development and repair. Since a parallel can be drawn with the ovulatory process in which multiple ECM proteins and ECM modifying enzymes are induced (Table 2), we suggest that CRISPLD2 may interact with these proteins and modifying enzymes during ovulation. Interestingly, CRISPLD2 is regulated by progesterone (P4) and its receptor (PGR) in the uterus, its expression is high during decidualization, constitutive during pregnancy and is dysregulated in patients with endometriosis [49]. A similar P4/PGR-dependent regulation of CRISPLD2 gene expression has been observed in rat granulosa cells [50]. However, despite the fact that the bovine CRISPLD2 proximal promoter does not harbor a PGR response element, the effect could be indirect as PGR is known to enhance functional activity of SP1 and GC-rich regions [51, 52], which are present in the bovine proximal promoter. CRISPLD2 has also been identified as a potential inhibitory modulator of the inflammatory and immune response, since glucocorticoids and interleukin-1β (IL1-β) increase CRISPLD2 mRNA and protein expression in airway smooth muscle cells, and CRISPLD2 modulates the expression of IL1-β responsive inflammatory genes such as IL6 and IL8 [53, 54]. Considering that ovulation is an acute inflammatory reaction and that the LH surge induces IL1-β in GC (review: [55]), another role for CRISPLD2 during ovulation may be to limit the inflammatory reaction. Thus, through its action on ECM proteins and modifying enzymes or the control of the inflammatory reaction, we propose that the induction of CRISPLD2 in GC of OF likely plays a key role in follicular rupture and ECM remodeling. Since CRISPLD2 knockout mice die during embryogenesis [56], a tissue-specific conditional knockout approach may be more appropriate to ultimately document the significance of ovarian CRISPLD2 gene expression.

Expression of *L3MBTL3* mRNA in GC was 7.5-fold stronger in OF as compared to DF but this expression was extinguished in CL, suggesting a potential role for L3MBTL3 in LH surge/hCG-induced events, such as ovulation and initial luteinization. L3MBTL3 is known to interact with the polycomb group repressive complex 1 (PRC1) family of proteins that maintains genes in a transcriptionally repressive state by modification of the chromatin [review: 57-58]. L3MBTL3 localizes into foci of the DNA-rich regions of the nucleus, and is classified as a chromatin histone methyllysine reader protein [57]. Methyllysine acts as docking site for specific reader proteins that alter chromatin structure to control various cellular processes. Proteins containing MBT domain such as L3MBTL3, which holds three MBT domains, are known to dimerize and to selectively recognize sequence specific mono and dimethyl-lysine residues within histone tails [57, 58]. This allows to target chromatin regulatory complexes, such as PRC1, to appropriate genomic loci, and has been functionally link to repression of gene expression. Moreover, the roles of MBT proteins have been associated with regulation of mitosis, tumor suppression, maintenance of cell identity and body pattern during development [59, 60]. In addition to the three MBT domains, L3MBTL3 harbors a SAM domain known to confer protein-protein interaction features and to act as a scaffolding protein for homo- or hetero-dimerization [58, 61]. Interestingly, we have shown in the present study that bovine *L3MBTL3* mRNA is alternatively spliced in GC, as compared to the complete mRNA expressed in bovine endometrial cells. To our knowledge, this is the first study to report an alternatively spliced isoform of *L3MBTL3* in mammalian species that results in a truncated open reading frame. The missing last seven amino acids, ^774^KNSHNEL^780^, of L3MBTL3 isoform 2 are located within the carboxy-terminal end following the SAM domain (^706^S-K^770^) known to confer protein/protein interactions [61]. These last amino acids (^774^K-L^780^) are well conserved in all mammalian species, which likely indicates a fundamental function for these amino acids in the epigenetic control of gene expression. Taken together, we hypothesize that the increase of L3MBTL3 expression in GC of ovulatory follicles may be necessary to silence specific genes that would otherwise negatively impact ovulation and/or initiation of luteinization. Additional studies will be required to unravel the physiological role of L3MBTL3 in GC function, and to investigate the functional significance of the C-terminal truncated L3MBTL3 spliced variant.

## Conclusions

In this study, we have identified several genes whose transcripts levels were increased following hCG injection. While the role of many of these genes is well documented, the exact function of genes such as FKBP5, POSTN, CRISPLD2, L3MBTL3 and others (Table 2) in the ovulatory and luteinization processes remain to be fully explored.

## Additional file

Additional file 1: Figure S1. Analyses of the cDNA subtraction efficiency. PCR analysis was performed on the indicated samples using *PTGS2* or *CYP19A1* specific primers, as described under Materials and Methods. PCR product aliquots were collected at increasing numbers of PCR cycles as indicated. The *PTGS2* DNA fragment (418 bp) was detected following 13 PCR cycles in the OF-DF subtracted sample but not until 18 PCR cycles in the corresponding unsubtracted OF sample. The *CYP19A1* DNA fragment (520 bp) was detected following 13 PCR cycles in the DF-OF subtracted sample but not until 18 PCR cycles in the corresponding unsubtracted DF sample. *PTGS2* or *CYP19A1* were not detected in the DF or OF samples, respectively. **Figure S2.** Representative differential screening results by macroarrays of the OF-DF cDNA library. PCR-amplified cDNA fragments (OF-DF) obtained by SSH were dot-blotted to generate two identical sets of membranes. A total of 940 individual cDNAs were dot-blotted. The macroarrays were then hybridized with two different probe set: subtracted OF-DF cDNAs (**A**), and reverse subtracted DF-OF cDNAs (**B**), as described under Materials and Methods. The two upper left hand dots for each membrane served as internal hybridization controls: A1 = *CYP19A1* (negative control) and A2 = *PTGS2* (positive control) for the OF-DF reaction. The cDNA clones that were found to be differentially expressed in the OF-DF membrane following comparison of hybridization signals among the corresponding spots of the two membranes were further characterized by sequencing. (PDF 275 kb)

## Abbreviations

CL: Corpus luteum
DF: Dominant follicles
eCG: equine chorionic gonadotropin
ECM: Extracellular matrix
GC: Granulosa cells
hCG: Human chorionic gonadotropin
LH: Luteinizing hormone
OF: Ovulatory follicles
OF-DF or DF-OF: Granulosa cell subtracted cDNA library
SF: Small follicles
SSH: Suppression subtractive hybridization
