# Supplementary material for: Gene expression profiling of upregulated mRNAs in granulosa cells of bovine ovulatory follicles following stimulation with hCG
Source: Reprod Biol Endocrinol. 2017 Nov 3;15:88. doi: 10.1186/s12958-017-0306-x (PMC5670713; doi:10.1186/s12958-017-0306-x)
Supplement: Additional file 1: Figure S1. — Analyses of the cDNA subtraction efficiency. PCR analysis was performed on the indicated samples using PTGS2 or CYP19A1 specific primers, as described under Materials and Methods. PCR product aliquots were collected at increasing numbers of PCR cycles as indicated. The PTGS2 DNA fragment (418 bp) was detected following 13 PCR cycles in the OF-DF subtracted sample but not until 18 PCR cycles in the corresponding unsubtracted OF sample. The CYP19A1 DNA fragment (520 bp) was detected following 13 PCR cycles in the DF-OF subtracted sample but not until 18 PCR cycles in the corresponding unsubtracted DF sample. PTGS2 or CYP19A1 were not detected in the DF or OF samples, respectively. Figure S2. Representative differential screening results by macroarrays of the OF-DF cDNA library. PCR-amplified cDNA fragments (OF-DF) obtained by SSH were dot-blotted to generate two identical sets of membranes. A total of 940 individual cDNAs were dot-blotted. The macroarrays were then hybridized with two different probe set: subtracted OF-DF cDNAs (A), and reverse subtracted DF-OF cDNAs (B), as described under Materials and Methods. The two upper left hand dots for each membrane served as internal hybridization controls: A1 = CYP19A1 (negative control) and A2 = PTGS2 (positive control) for the OF-DF reaction. The cDNA clones that were found to be differentially expressed in the OF-DF membrane following comparison of hybridization signals among the corresponding spots of the two membranes were further characterized by sequencing. (PDF 275 kb) [file 12958_2017_306_MOESM1_ESM.pdf]

SUPPLEMENTARY FIGURE LEGENDS

Supplementary FIG. 1.

Analyses of the cDNA subtraction efficiency. PCR analysis was performed on the indicated samples using *PTGS2* or *CYP19A1* specific primers, as described under Materials and Methods. PCR product aliquots were collected at increasing numbers of PCR cycles as indicated. The *PTGS2* DNA fragment (418 bp) was detected following 13 PCR cycles in the OF-DF subtracted sample but not until 18 PCR cycles in the corresponding unsubtracted OF sample. The *CYP19A1* DNA fragment (520 bp) was detected following 13 PCR cycles in the DF-OF subtracted sample but not until 18 PCR cycles in the corresponding unsubtracted DF sample. *PTGS2* or *CYP19A1* were not detected in the DF or OF samples, respectively.

Supplementary FIG. 2.

Representative differential screening results by macroarrays of the OF-DF cDNA library. PCR-amplified cDNA fragments (OF-DF) obtained by SSH were dot-blotted to generate two identical sets of membranes. A total of 940 individual cDNAs were dot-blotted. The macroarrays were then hybridized with two different probe set: subtracted OF-DF cDNAs (**A**), and reverse-subtracted DF-OF cDNAs (**B**), as described under Materials and Methods. The two upper left-hand dots for each membrane served as internal hybridization controls: A1 = *CYP19A1* (negative control) and A2 = *PTGS2* (positive control) for the OF-DF reaction. The cDNA clones that were found to be differentially expressed in the OF-DF membrane following comparison of hybridization signals among the corresponding spots of the two membranes were further characterized by sequencing.

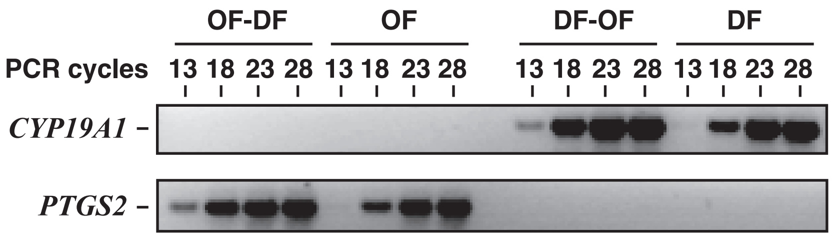

Supplementary Figure 1

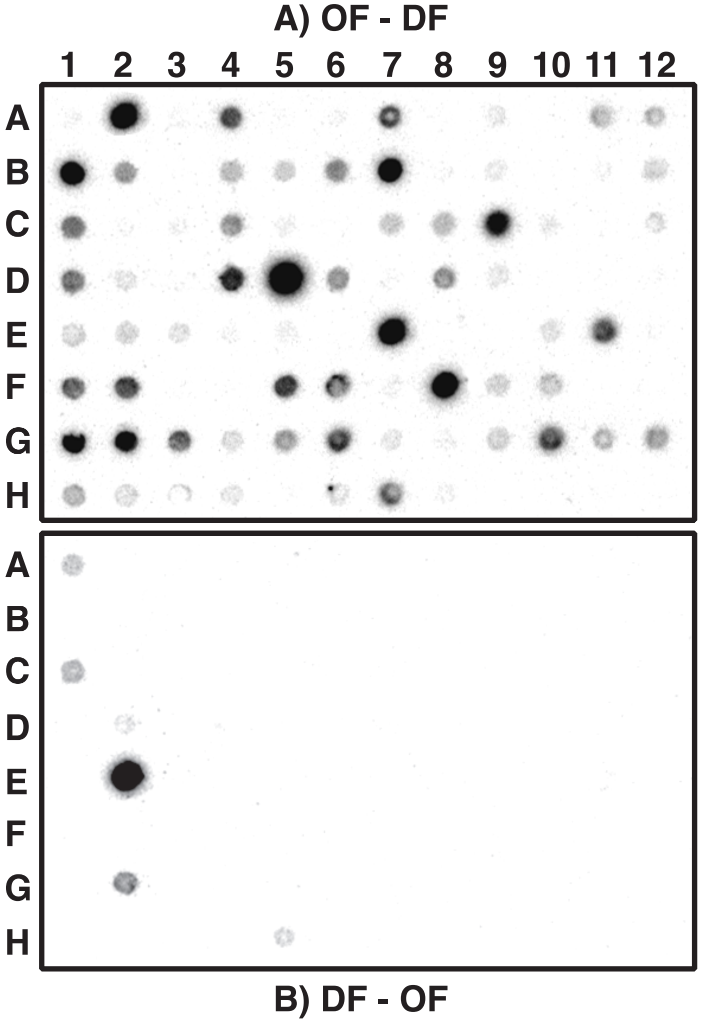

Supplementary Figure 2
